# Effectiveness of a Group B Outer Membrane Vesicle Meningococcal Vaccine in Preventing Hospitalization from Gonorrhea in New Zealand: A Retrospective Cohort Study

**DOI:** 10.3390/vaccines7010005

**Published:** 2019-01-05

**Authors:** Janine Paynter, Felicity Goodyear-Smith, Jane Morgan, Peter Saxton, Steven Black, Helen Petousis-Harris

**Affiliations:** 1 Department of General Practice and Primary Health, University of Auckland, Auckland 1142, New Zealand; f.goodyear-smith@auckland.ac.nz; 2 Sexual Health Services Waikato District Health Board and Honorary Senior Lecturer, School of Medicine, University of Auckland, Auckland 1142, New Zealand; Jane.Morgan@waikatodhb.health.nz; 3 Department of Social and Community Health, University of Auckland, Auckland 1142, New Zealand; p.saxton@auckland.ac.nz; 4 Department of Pediatrics, Cincinnati Children’s Hospital, Cincinnati, OH 45229-3039, USA; stevblack@gmail.com; 5 Immunisation Advisory Centre, Department of General Practice and Primary Health Care, University of Auckland, Auckland 1142, New Zealand; h.petousis-harris@auckland.ac.nz

**Keywords:** gonorrhea, outer membrane vesicle vaccine, group B meningococcus, cohort study, New Zealand

## Abstract

Gonorrhea is a major global public health problem with emergence of multiple drug-resistant strains with no effective vaccine. This retrospective cohort study aimed to estimate the effectiveness of the New Zealand meningococcal B vaccine against gonorrhea-associated hospitalization. The cohort consisted of individuals born from 1984 to 1999 residing in New Zealand. Therefore, it was eligible for meningococcal B vaccination from 2004 to 2008. Administrative datasets of demographics, customs, hospitalization, education, income tax, and immunization were linked using the national Integrated Data Infrastructure. The primary outcome was hospitalization with a primary diagnosis of gonorrhea. Cox’s proportional hazards models were applied with a Firth correction for rare outcomes to generate estimates of hazard ratios. Vaccine effectiveness estimates were calculated as 1-Hazard Ratio expressed as a percentage. There were 1,143,897 eligible cohort members with 135 missing information on gender, 16,245 missing ethnicity, and 197,502 missing deprivation. Therefore, only 935,496 cohort members were included in the analysis. After adjustment for gender, ethnicity, and deprivation, vaccine effectiveness (MeNZB™) against hospitalization caused by gonorrhea was estimated to be 24% (95% CI 1–42%). In conclusion, the data suggests vaccination with MeNZB™ significantly reduced the rate of hospitalization from gonorrhea. This supports prior research indicating possible cross protection of this vaccine against gonorrhea acquisition and disease in the outpatient setting.

## 1. Introduction

Gonorrhea is a major international public health problem [1,2] that is now exacerbated by the increasing emergence of multiple drug-resistant strains [3,4,5]. To date, development of an effective vaccine has been unsuccessful [6]. Contracting the infection does not provide immunity and, hence, repeated infections can commonly occur [7]. Unfortunately, there is no known immunological correlate of protection to guide vaccine development.

Since gonorrhea is a reportable disease in most developed countries, the incidence trends over time are relatively easy to track. Using ecological data from national disease surveillance reports, a decline in gonorrhea in the period immediately following use of group B meningococcal outer membrane vesicles (OMV) vaccines in Cuba [8], New Zealand (NZ) [9], and, to a limited extent, Norway [10] suggested that meningococcal B OMV vaccines may reduce the risk of gonorrhea. The 80% to 90% genetic homology between *Neisseria meningitidis* and *N. gonorrhoeae* offers a biologically plausible mechanism by which cross protection might occur [11].

In New Zealand, 81% of the population aged 0 to 20 years received doses of the strain-specific OMV meningococcal B (MeNZB™) vaccine from 2004 through schools and primary care, in response to a prolonged epidemic of group B meningococcal disease [12]. The mass immunization program ran for two years from 2004 to 2006, and the vaccine was available until 2008. Approximately one million infants, children, and young people received all three doses of the vaccine. In NZ, everyone is assigned a National Health Index number (NHI). This unique person–specific alphanumeric identifier is used across all health systems and makes data linkage relatively simple and robust.

This infrastructure enabled us to conduct a case-control study using immunization and sexual health clinic data to evaluate the vaccine effectiveness (VE) of the 3+0 (three primary doses, no booster) schedule of MeNZB™, used in NZ from 2004 to 2008, against confirmed gonorrhea cases between 2004 and 2016 in young adults aged 15 to 30 years. In this study, there were 14,730 cases and controls for analyses consisting of 1241 incidences of gonorrhea, 12,487 incidences of chlamydia, and 1002 incidences of co-infection. We were able to determine that those vaccinated were less likely to become infected with 41% vs. 49%, adjusted OR 0.69 (95%CI 0.61–0.79). After adjusting for ethnicity, deprivation (socio-economic status), geographic area, and gender, we found a VE of 31% (95% CI 21–39) [13].

In addition to cases presenting at sexual health clinics and primary care, gonorrhea may also be associated with significant morbidity including pelvic inflammatory disease, ectopic pregnancy, infertility, and chronic pain. An Australian retrospective cohort study found 5% (45 out of 1015) women diagnosed with gonorrhea were subsequently hospitalized with pelvic inflammatory disease, whereas only 1.3% (483/38,193) with chlamydia were hospitalized [14]. Similarly, gonorrhea may result in hospitalization for orchitis or epididymitis in men. 

We wished to explore the effectiveness of the MeNZB™ vaccine in reducing hospitalizations from gonorrhea in the same study population as our case-control study. Our aim was to estimate the VE of the 3+0 immunization series against gonorrhea-associated hospitalization using a retrospective cohort study.

## 2. Materials and Methods 

This is a retrospective cohort study design.

### 2.1. Study Population

The study population includes New Zealand residents born in 1984 to 1999 inclusively and were residing in New Zealand from 2004 (the start of the MeNZB program) until at least 2015. Cohort eligibility (i.e., residence in New Zealand) was determined using the NHI demographic dataset. Customs New Zealand journey information (all arrivals and departures via New Zealand ports from late 1997, secondary and tertiary education data, the National Immunisation Register (NIR), and a restricted subset of income tax data records). Record linkage was completed through use of the Integrated Data Infrastructure (IDI) provided by Statistics New Zealand [15]. Deaths prior to 2004, and those who were absent from New Zealand from or before January 2004, as indicated by travel data or a lack of presence in the education, tax, health, and address notifications datasets were excluded from the study. Logic checks were done to remove mismatched linked data (for example, individuals with travel dates occurring earlier than their birth date). Participants for this study were selected from the same population as the earlier case-control study.

### 2.2. Vaccination Status and Other Descriptive Variables

Cohort members were considered fully vaccinated if they had received three doses of the MeNZB™ vaccine. Members who had one or two doses were considered partially vaccinated. Information on vaccine doses and dates was obtained from the National Immunisation Register.

Ethnicity (priority coded Māori, Pacific Island, and New Zealand European/Asian/other) and gender are provided in the NHI dataset. Where available, deprivation is estimated using each cohort members’ most recent residential mesh block and the New Zealand deprivation index [16]. A value of 1 indicates a mesh block is in the 10% least deprived areas in New Zealand. A value of 10 is the 10% most deprived. The deciles are collapsed in this study to three groups of low, medium, and high deprivation. Deprivation provides an indication of a socio-economic status. Low deprivation indicates that the individual is likely to have a higher socio-economic status and a high deprivation indicates a lower socio-economic status.

The risk of contracting gonorrhea is associated with sexual activity. Therefore, the likely age of sexual debut impacts the risk of gonorrhea and other sexually transmitted diseases. There was a wide age range (4–20 years) among our cohort at the start of the vaccination program in 2004. The reported average age for sexual debut in NZ is 15–16 years with very few young people reporting sexual activity before 13 years. Therefore, the age at which each cohort member turns 13 was chosen as the start date for follow-up in our study.

### 2.3. Outcome Variables/Events

The primary outcome considered in this analysis is hospitalization with gonorrhea, where gonorrhea is considered the reason for hospitalization of cohort members. Table 1 shows the specific ICD-10-AM codes used. Selection of the diagnosis codes was made by members of the team with clinical experience in the area of sexually transmitted disease and broader consultation with clinicians working in this area. Group 1 is the most specific because these codes identify gonococcal bacteria as the cause. The hospital coding is very unlikely to have specified gonorrhea without laboratory verification and New Zealand has a high proportion of testing suspected cases (30–50% of youth) [17]. Prior to accessing the data, we were unsure how many outcomes there would be that were specifically attributed to gonorrhea. Therefore, we decided a priori on a set of conditions that may be caused by gonorrhea. Group 2 were genitourinary infections, which, based on our clinical experience and knowledge of the literature, may be frequently caused by gonorrhea as well as by other organisms (such as chlamydia). Group 3 are conditions with a range of causative agents for which gonorrhea-attributable cases will be a small minority.

### 2.4. Statistical Analysis

Descriptive statistics (frequencies and percentages) are presented for each of the main variables of interest. In order to meet the privacy protection requirements of Statistics New Zealand, each count (both population and numbers of cases) has been randomly rounded to a base of 3. Percentages are based on rounded counts. Counts with original values <6 are suppressed and identified in this report as S.

Cox’s proportional hazards models (proc PHREG as per SAS Enterprise Guide v7.1 (SAS Institute Inc, Cary, NC, USA) were applied with a Firth correction for rare outcomes and predictive covariates in order to generate estimates of hazard ratios and robust risk limits. The vaccination status was modeled as a time–dependent variable. Individuals who did not receive any doses of MeNZB™ were unvaccinated for all of the follow-up visits. Individuals who had received three doses prior to their 13th birthday were vaccinated for all of the follow-up visits. Those who had received one or two doses before the follow-up were partially vaccinated. If unvaccinated before the follow-up, then the status changed on receipt of the first dose from unvaccinated to partial vaccination. Receipt of a third dose during follow-up changed the status from partial to vaccinated. Univariate and multivariable model estimates are presented. Cohort members were censored if they died or left New Zealand for longer than 6 weeks after the start of the follow-up (13^th^ birthday) before hospitalization or the end of the study period on 31 December, 2015. This means that follow up time varied from 3 to 18 years depending on the year of birth. An absence of up to six weeks was permitted in order to minimize the bias that may occur from a cohort member being hospitalized overseas, and, therefore, not recorded in our data, but allow for the relatively common practice for young New Zealanders of an overseas vacation. Unrounded data was used as the model input. The VE estimates are calculated as a 1-Hazard Ratio and expressed as a percent. The unit of follow up time was set as groups of 30 days (approximation of months) from the start date (13th birthday). 

Ethical approval (approval reference 15/CEN/189).was obtained from New Zealand’s Health and Disability Ethics Committee.

## 3. Results

Our primary outcome was defined as a hospitalization specifically due to gonorrhea infection. There were 1,143,897 eligible cohort members of NZ residents born between 1984 and 1999 inclusively residing in NZ from the age of 13 years including from 2004 through 2015. There were 135 individuals missing information on sex, 16,245 missing ethnicity and/or 197,502 missing deprivation. Therefore, 935,496 individuals, with data on sex, ethnicity, and deprivation were included in the analysis. Just under half of the cohort (48.8%) were female. Europeans were the largest ethnic group, and were combined with the two other smallest groups including other and Asian, which form 62.3% of the cohort. Māori were a quarter of the cohort (25.7%) and Pacific Island ethnicities made up the remaining 10% of the cohort. Furthermore, 21% of the cohort lived in an area of low deprivation with about 30% of medium deprivation, and another 30% in an area of high deprivation. The largest proportion of missing data was due to deprivation (17%).

Table 2 shows a demographic breakdown of the cohort and vaccination status. There was only a minor difference in vaccination coverage by gender. Māori found that Europeans, other ethnicities, and Pacific Peoples were more likely to be vaccinated. Vaccination coverage was similar across deprivation levels. The middle and younger-aged members of the cohort were more likely to be vaccinated with only 32% of those born between 1984 and 1988 vaccinated, compared to 74% of those born between 1989 and 1993 vaccinated, and 77% of those born from 1994 to 1999.

There were 261 cases of first hospitalization attributable to gonorrhea specifically, which makes this a rare outcome. Hospitalizations (5124) with a group 2 condition were the most common. Lastly, there were 3225 hospitalizations in the group 3 categories. For analysis outcomes, these hospitalizations combined as follows: outcome one was a hospitalization specifically attributable to gonorrhea. Outcome 2 was a hospitalization specifically attributable to gonorrhea or a group 2 condition so the combined number of outcomes was 5385. Outcome 3 was a combination of hospitalizations specifically attributable to gonorrhea, group 2, and group 3 conditions, which totals 8610. The outcomes 1–3 decrease in specificity.

Hospitalized cases were more likely to be female for outcomes one, two, and three (Table 3, Table 4 and Table 5). Those in the highest deprivation were also more likely to be hospitalized for all three outcomes. However, Māori were notably much more likely with Hazard Ratio = 8 (6–12 95% CI) to be hospitalized with a gonorrhea-specified outcome compared to NZ Europeans and other (Table 3). There was less disparity between Māori and NZ Europeans and others when considering group 2 and group 3 outcomes (Table 4 and Table 5).

Vaccinated individuals were significantly less likely to be hospitalized due to gonorrhea after adjusting for gender, ethnicity, and deprivation (HR 0.76, 95% CI 0.58–0.99). The results show a small to medium effect size. This gives a vaccine effectiveness estimate of 24% (95% CI 1–42%). We conducted a sensitivity analysis looking at three subgroups within the cohort based on age. The middle subgroup of the cohort who were vaccinated as young teenagers and, therefore, were most likely to have had the vaccination shortly before sexual activity commenced (median age 13). The estimated vaccine effectiveness for this group was 47% (95%CI 18–66%). There was no significant measurable vaccine effect in the youngest (median age 8) or oldest cohort subgroups (median age 18).

Vaccination was significantly associated with an increased risk of hospitalization (HR 1.28, 95% CI 1.21–1.36 for outcome 2, HR 1.34, 95% CI 1.28–1.41 for outcome 3) when outcomes included a range of conditions likely to be caused by other sexually transmitted organisms such as chlamydia, which is more common than gonorrhea. This also had a small to medium effect.

## 4. Discussion

Vaccines may protect against infection as well as prevent disease and/or mitigate disease severity [13,18,19]. We found evidence that MeNZB™ may be moderately effective at preventing hospitalization specifically attributable to gonorrhea with an estimated vaccine effectiveness of 24%. This is a slightly lower vaccine effectiveness estimate for preventing cases of gonorrhea presenting at sexual health clinics (31%) [13].

When considering hospitalization attributable to sexually transmitted disease more broadly (outcomes two and three), the study found an increased risk (small effect) of hospitalization among vaccinated individuals. These outcomes were much less specific and there is likely to be a number of factors, including bias due to misclassification, i.e., sexually transmitted diseases not due to gonorrhea, which contribute to this finding. However, the most likely explanation is that our predominantly unvaccinated older cohort were in the study for a longer period. The end of follow-up was the date up to which we had data on hospitalizations in order to maximize the number of cases (hence power) of a rare outcome. The risk of acquiring a sexually transmitted infection is lower after 24 years of age. The older members of the cohort would have reached and been beyond this and they were more likely to be unvaccinated and at lower risk. In addition, for the younger cohort members, there is more time elapsed between vaccination and risk of a sexually transmitted infection so there may be a waning of the vaccine effect. Our sensitivity analysis failed to detect a significant impact on the younger cohort members, which suggests that the vaccine effect may have waned after four years, although the sample size for this younger group means there was limited power to detect lower levels of a vaccine effect.

What is important about the difference in the effect of vaccination for the outcome 1 (gonorrhea specific) versus more non-specific outcomes 2 and 3, is that outcomes 2 and 3 provide a reasonable broader measure of risk of hospitalization due to sexually transmitted infection in the vaccinated versus unvaccinated population. This strengthens the case for an effect of the vaccine per se and not an artefact of confounding due to a difference in risky sexual behaviors between vaccinated versus unvaccinated found in both this study and our earlier case-control study drawn from the same population.

A detailed genomic investigation of *N. gonorrhoeae* in New Zealand was carried out from October 2014 to May 2015, but, prior to this, there were no studies examining the genomic epidemiology of gonorrhoea in the Western Pacific [1]. There is no available dataset in NZ that provides detail on the strain of gonorrhoea isolated from a case that we could link to our study cohort. Future reporting could include the sero epidemiology of gonorrhoea in the period prior to and during the MeNZB campaign and examine these in relation to the recent report [20]. 

Based on DNA-DNA hybridisation, the homology of primary sequences between *N. meningititis* and *N. gonorrhoeae* is between 80% and 90%. *N. meningitidis* OMV vesicles provide many potential antigens that may be cross reactive against *N. gonorrhoeae* including a phospholipid bilayer with over 200 proteins such as the porin A (PorA), porin B (porB), reduction-modifiable protein (Rmp), opacity-associated proteins (Opc and Opa), *Neisseria* surface protein A (NspA), and lipopolysaccharide (LPS). The lumen includes a variety of periplasmic constituents [21]. While the most abundant proteins are the PorA, PorB, RmP, and OpcA, the NspA, FbpA, FetA, Omp85, and TdfH were also present [22].

Some of the many meningococcal potential epitopes and functional immune responses have been demonstrated against more conserved regions of PorA, PorB, OpcA, Omp85, and LPS [20]. More recently, immunogenicity was demonstrated against Rmp, Opa, FbpA, and less well known antigens exopolyphosphatase and gamma-glutamyltranspeptidase and a cell binding factor NMBO345 [23,24,25]. There will likely be others [24]. Of importance will be the homologous equivalents in *N. gonorrhoeae* of which a number of possibilities have been observed [25]. The potential homologous epitopes between OMV antigens and *N. gonorrhoeae* is an area warranting further investigation.

In addition to the NZ OMV, Bexsero® includes three recombinant proteins (NHBA, fHbp, and NadA) that are variably shared with *N. gonorrhoeae*, although NadA is absent in all *N. gonorrhoeae* studied thus far [26,27]. Therefore, there is a possibility that this vaccine may provide similar protection to the MeNZB™ vaccine and we recommend this as a priority area for further research.

### 4.1. Strengths

We were able to link a number of datasets using the unique NHI number to establish a cohort that met our eligibility criteria. The use of large administrative datasets enables us to capture a large proportion (almost all) of the New Zealand population and, therefore, investigate the impact of the vaccine on a rarer, more severe outcome. 

### 4.2. Limitations

Hospitalizations due to gonorrhea represent only the very severe end of the disease spectrum and are rare. It means that the vaccine effectiveness estimate provided by a Cox’s proportional hazard regression may be overestimated. However, we corrected for this possibility using a recommended method.

We have assumed age as an indicator of commencement of sexual activity and, therefore, the risk of getting a sexually transmitted infection. The start of follow up was set at 13 years. However, there are likely to have been many who were not sexually active and, therefore, not at risk until much older, e.g., 18 years. This is only of concern if there is likely to be a strong association between the age of onset of sexual activity and likelihood of vaccination. Given that the vaccination program was school-based and achieved good coverage, including for groups perceived at a higher risk of poor outcomes, this concern is unlikely.

The rarity of the outcome also makes it difficult to draw meaningful inferences about vaccine effectiveness for specific subgroups such as Māori.

In our case control study, we found a 31% VE against gonococcal infection. This current cohort study suggests that MeNZB™ may afford 24% VE against hospitalization for gonorrhea. Gonorrheal infection is a spectrum ranging from very mild or asymptomatic to a serious systemic and life-threatening disease. It is likely that, in some cases, the vaccine does not completely prevent infection, but provides sufficient cross-protection so that only a mild case of the disease is experienced.

This was an observational study using data that was not collected with the purpose of investigating the impact of the MeNZB™ on gonorrhea. While we have incorporated data on common confounders, there may still be unknown confounding influences on the outcome.

Protection against gonococcal infection would impart considerable public health benefits, particularly with the global increase in antimicrobial resistant strains. Our findings lend further weight to the premise that it should be possible to develop an OMV, subunit, or combination of a gonorrhea-specific vaccine.

## Figures and Tables

**Table 1:** ICD-10 codes used to identify primary (main reason for) hospitalizations of interest.

ICD10 Code	Text Description	Gonorrhea Only	Outcome 2	Outcome 3
A54.0	Gonococcal infection including cervicitis, cystitis, urethritis, vulvovaginitis	Yes	Yes	Yes
A54.2	Gonococcal pelviperitonitis including epididymitis, female pelvic inflammatory disease, orchitis, prostatitis	Yes	Yes	Yes
A54.3	Gonococcal conjunctivitis	Yes	Yes	Yes
A54.4	Gonococcal infection of musculoskeletal system	Yes	Yes	Yes
A54.8	Other gonococcal infections including meningitis, septicaemia	Yes	Yes	Yes
A54.9	Gonococcal infection, unspecified	Yes	Yes	Yes
O98.2	Gonorrhea complicating pregnancy	Yes	Yes	Yes
N74.3	Female gonococcal pelvic inflammatory disease	Yes	Yes	Yes
N41.0	Acute prostatitis	No	Yes	Yes
N41.2	Abscess of prostate	No	Yes	Yes
N41.9	Inflammatory disease of prostate, unspecified	No	Yes	Yes
N45.0	Orchitis, epididymitis, and epididymo-orchitis with abscess	No	Yes	Yes
N45.9	Orchitis, epididymitis, and epididymo-orchitis without abscess	No	Yes	Yes
N70.0	Acute salpingitis and oophoritis	No	Yes	Yes
N70.1	Chronic salpingitis and oophoritis	No	Yes	Yes
N70.9	Salpingitis and oophoritis, unspecified	No	Yes	Yes
N71.0	Acute inflammatory disease of uterus	No	Yes	Yes
N71.1	Chronic inflammatory disease of uterus	No	Yes	Yes
N71.9	Inflammatory disease of uterus, unspecified	No	Yes	Yes
N72.0	Inflammatory disease of cervix uteri	No	Yes	Yes
N73.0	Acute parametritis and pelvic cellulitis	No	Yes	Yes
N73.1	Chronic parametritis and pelvic cellulitis	No	Yes	Yes
N73.2	Parametritis and pelvic cellulitis, unspecified	No	Yes	Yes
N73.8	Other specified female pelvic inflammatory disease	No	Yes	Yes
N73.9	Female pelvic inflammatory disease, unspecified	No	Yes	Yes
J02.9	Acute pharyngitis	No	No	Yes
K62.8	Other specified diseases of anus and rectum, proctitis NOS	No	No	Yes
N41.1	Chronic prostatitis	No	No	Yes
N48.2	Other inflammatory diseases of penis	No	No	Yes
N73.3	Female acute pelvic peritonitis	No	No	Yes
N73.5	Female pelvic peritonitis, unspecified	No	No	Yes
N73.6	Female pelvic peritoneal adhesions	No	No	Yes
N75.0	Cyst of Bartholin’s gland	No	No	Yes
N75.1	Abscess of Bartholin’s gland	No	No	Yes
N75.8	Other diseases of Bartholin’s gland	No	No	Yes
N75.9	Disease of Bartholin’s gland, unspecified	No	No	Yes
N76.0	Acute vaginitis	No	No	Yes
N76.1	Subacute and chronic vaginitis	No	No	Yes
N76.2	Acute vulvitis	No	No	Yes
N76.3	Subacute and chronic vulvitis	No	No	Yes
N76.4	Abscess of vulva	No	No	Yes
N76.5	Ulceration of vagina	No	No	Yes
N76.6	Ulceration of vulva	No	No	Yes

**Table 2:** Associations between co-variates and the vaccination status.

Co-Variate	Partially Vaccinated	(%)	Unvaccinated	(%)	Vaccinated	(%)	Total ^1^
**Gender**							
Female	5343	(1.0)	225,243	(40.4)	327,576	(58.7)	558,162
Male	6204	(1.1)	241,338	(41.2)	338,061	(57.7)	585,600
**Ethnicity**							
European & Other	5082	(0.7)	308,016	(42.9)	405,606	(56.4)	718,704
Māori	4806	(1.6)	91,767	(31.3)	196,959	(67.1)	293,529
Pacific Peoples	1584	(1.4)	51,768	(44.9)	62,067	(53.8)	115,422
**Deprivation**							
Low	1932	(0.8)	86,910	(36.7)	148,143	(62.5)	236,985
Medium	3075		138,690	(38.7)	216,315	(60.4)	358,080
High	4710	(1.3)	137,940	(39.3)	208,671	(59.4)	351,321
**Hospitalization**							
None	11,397	(1.0)	463,272	(40.8)	660,615	(58.2)	1,135,287
Gonorrhea only	S ^2^		132	(50.6)	S		261
Group 2 only	90	(1.8)	2037	(39.8)	2997	(58.5)	5124
Group 3 only	S		1266	(39.3)	S		3225

^1^ Counts have been randomly rounded to base 3 as per Statistics NZ to protect privacy and confidentiality and may not reconcile precisely. ^2^ S indicates that this value has been suppressed as per Statistics NZ to protect privacy and confidentiality. A relatively small proportion (1%) of the cohort was partially vaccinated (about 11,547) compared to those either unvaccinated (41%) or vaccinated (58%). This group of partially vaccinated individuals also had a higher proportion of Māori and those with the highest deprivation compared to unvaccinated and vaccinated groups.

**Table 3:** Cox’s proportional hazards with Firth correction for rare events estimates and 95% confidence intervals (CI) for the primary outcome (hospitalization with gonorrhea was specified).

Characteristic	Hospitalization with Gonorrhea				
	Yes	%	Total	HR	Adjusted HR
**Gender**					
Missing	S	S	135	NA	NA
Female	213	(0.038)	558,162		Ref
Male	48	(0.008)	585,603		0.22 (0.16–0.30)
**Ethnicity**					
Missing	S	S	16,245		
European & other	45	(0.006)	718,704		Ref
Māori	198	(0.067)	293,529		8.44 (5.87–12.13)
Pacific Peoples	18	(0.016)	115,422		2.04 (1.12–3.73)
**Deprivation**					
Missing	36	(0.018)	197,511		NA
High	144	(0.041)	351,321		2.30 (1.42–3.75)
Medium	63	(0.018)	358,080		1.55 (0.93–2.57)
Low	18	(0.008)	236,982		Ref
**Vaccination Status**					
Unvaccinated	129	(0.028)	466,710		Ref
Partial	S	S	S	0.42 (0.08–2.12)	0.31 (0.06–1.57)
Vaccinated	132	(0.019)	677,190	0.89 (0.69–1.16)	0.76 (0.58–0.99)
**Total**	261	(0.023)	1,143,897		

**Table 4:** Cox’s proportional hazards with Firth correction for rare events estimates and 95% confidence intervals (CI) for group 2 outcomes.

Characteristic	Hospitalization with Gonorrhea or a Group 2 Diagnosis				
	Yes	%	Total	HR	Adjusted HR
**Gender**					
Missing	S	S	135	NA	NA
Female	3945	0.71	558,162		Reference
Male	1443	0.25	585,600		0.34 (0.32–0.36)
**Ethnicity**					
Missing	S	S	16,245	NA	NA
European & Other	2202	0.31	718,704		Reference
Māori	2652	0.90	293,529		2.45 (2.29–2.61)
Pacific Peoples	531	0.46	115,422		1.41 (1.27–1.57)
**Deprivation**					
Missing	729	0.37	197,511	NA	NA
High	2319	0.66	351,321		1.40 (1.29–1.53)
Medium	1578	0.44	358,083		1.15 (1.05–1.25)
Low	762	0.32	236,985		Ref
**Vaccination status**					
Unvaccinated	2169	0.46	466,710		Reference
Partial	96	0.83	11,547	1.65 (1.32–2.01)	1.45 (1.16–1.82)
Vaccinated	3123	0.47	665,640	1.36 (1.29–1.45)	1.28 (1.21–1.36)
**Total**	5385	0.47	1,143,897		

**Table 5:** Cox’s proportional hazards with Firth correction for rare event estimates and 95% confidence intervals (CI) for group 3 outcomes.

Characteristic	Hospitalization with Gonorrhea, Group 2, or Group 3 Diagnosis				
	Yes	%	Total	HR	Adjusted HR
**Gender**					
Missing	S	S	135		NA
Female	6579	1.18	558,162		Reference
Male	2031	0.35	585,600		0.28 (0.27–1.41)
**Ethnicity**					
Missing	S	S	16,245		NA
European & Other	3936	0.55	718,704		Reference
Māori	3828	1.30	293,529		2.00 (1.90–2.10)
Pacific Peoples	846	0.73	115,422		1.28 (1.18–1.39)
**Deprivation**					
Missing	1173	0.59	197,511		NA
High	3525	1.00	351,321		1.37 (1.29–1.47)
Medium	2640	0.74	358,080		1.18 (1.10–1.26)
Low	1272	0.54	236,985		Reference
**Vaccination status**					
Unvaccinated	3438	0.74	466,710		Reference
Partial	150	1.30	11,547	1.64 (1.37–1.96)	1.49 (1.24–1.78)
Vaccinated	5025	0.75	665,640	1.41 (1.35–1.48)	1.34 (1.28–1.41)
**Total**	8610	0.75	1,143,897		

## Disclaimer

The results in this paper are not official statistics. The data have been created for research purposes from the Integrated Data Infrastructure (IDI), managed by Statistics New Zealand. The opinions, findings, recommendations, and conclusions expressed in this paper are those of the author, not Statistics New Zealand. Statistics New Zealand provided access to the anonymized data used in this study in accordance with security and confidentiality provisions of the Statistics Act 1975. Only people authorized by the Statistics Act 1975 are allowed to see data about a particular person, household, business, or organization, and the results in this paper have been made confidential to protect these groups from identification. Careful consideration has been given to the privacy, security, and confidentiality issues associated with using administrative and survey data in the IDI. Further detail can be found in the Privacy impact assessment for the Integrated Data Infrastructure available from www.stats.govt.nz.
